# Predicting quetiapine dose in patients with depression using machine learning techniques based on real-world evidence

**DOI:** 10.1186/s12991-023-00483-w

**Published:** 2024-01-06

**Authors:** Yupei Hao, Jinyuan Zhang, Jing Yu, Ze Yu, Lin Yang, Xin Hao, Fei Gao, Chunhua Zhou

**Affiliations:** 1 Department of Clinical Pharmacy, the First Hospital of Hebei Medical University, Shijiazhuang, China; 2 The Technology Innovation Center for Artificial Intelligence in Clinical Pharmacy of Hebei Province, The First Hospital of Hebei Medical University, Shijiazhuang, China; 3 Beijing Medicinovo Technology Co., Ltd, Beijing, China; 4 Dalian Medicinovo Technology Co., Ltd, Dalian, China

**Keywords:** Quetiapine, Machine learning, Dose, Prediction model, Depression

## Abstract

**Background:**

Being one of the most widespread, pervasive, and troublesome illnesses in the world, depression causes dysfunction in various spheres of individual and social life. Regrettably, despite obtaining evidence-based antidepressant medication, up to 70% of people are going to continue to experience troublesome symptoms. Quetiapine, as one of the most commonly prescribed antipsychotic medication worldwide, has been reported as an effective augmentation strategy to antidepressants. The right quetiapine dose and personalized quetiapine treatment are frequently challenging for clinicians. This study aimed to identify important influencing variables for quetiapine dose by maximizing the use of data from real world, and develop a predictive model of quetiapine dose through machine learning techniques to support selections for treatment regimens.

**Methods:**

The study comprised 308 depressed patients who were medicated with quetiapine and hospitalized in the First Hospital of Hebei Medical University, from November 1, 2019, to August 31, 2022. To identify the important variables influencing the dose of quetiapine, a univariate analysis was applied. The prediction abilities of nine machine learning models (XGBoost, LightGBM, RF, GBDT, SVM, LR, ANN, DT) were compared. Algorithm with the optimal model performance was chosen to develop the prediction model.

**Results:**

Four predictors were selected from 38 variables by the univariate analysis (p < 0.05), including quetiapine TDM value, age, mean corpuscular hemoglobin concentration, and total bile acid. Ultimately, the XGBoost algorithm was used to create a prediction model for quetiapine dose that had the greatest predictive performance (accuracy = 0.69) out of nine models. In the testing cohort (62 cases), a total of 43 cases were correctly predicted of the quetiapine dose regimen. In dose subgroup analysis, AUROC for patients with daily dose of 100 mg, 200 mg, 300 mg and 400 mg were 0.99, 0.75, 0.93 and 0.86, respectively.

**Conclusions:**

In this work, machine learning techniques are used for the first time to estimate the dose of quetiapine for patients with depression, which is valuable for the clinical drug recommendations.

**Supplementary Information:**

The online version contains supplementary material available at 10.1186/s12991-023-00483-w.

## Background

Depression is a severe affective mental disorder that is accompanied by a lack of pleasure, and the impairment of cognition, behavior and autonomic nerve function, which causes dysfunction in various spheres of individual and social life, severely limits psychosocial functioning, and diminishes quality of life [1, 2]. Being one of the most widespread, pervasive, and troublesome illnesses in the world [3–5], depression can affect individuals of any age. By 2020, depression is anticipated to overtake heart disease as the second-leading cause of disability or early death, according to estimates from the World Health Organization (WHO) [6]. As a common and disabling mental disorder [7], it is a serious global public health issue that not only results in personal misery for those affected but also places a large economic burden on both the patients and the entire society [8, 9]. When it comes to medicinal therapy for depressive disorders, the American Psychiatric Association recommends selective serotonin reuptake inhibitors (SSRI, such as sertraline) and serotonin–norepinephrine reuptake inhibitors (SNRI, such as duloxetine), as well as noradrenergic and specific serotonergic antidepressants (NaSSA, such as mirtazapine) [10, 11]. Regrettably, despite obtaining evidence-based antidepressant medication, up to 70% of people are going to continue to experience troublesome symptoms [12, 13].

According to the Canadian Network for Mood and Anxiety Treatments (CANMAT) guidelines and American Psychiatric Association Practice guidelines, atypical antipsychotics (AA), specifically the use of quetiapine has been reported as an effective augmentation strategy to antidepressants. Quetiapine is an atypical antipsychotic agent, which was first introduced in the pharmaceutical market in 1997 [14]. In 2010, the European Medicine Agency (EMA) approved the extended-release formulation of the drug, quetiapine XR, as an add-on to antidepressants when monotherapy gives suboptimal response [15]. Studies have shown that quetiapine (mean dose, 156.74 ± 97.6 mg/day) showed significant benefits for both response and remission rates compared to placebo [16, 17]. Despite its high effectiveness, its optimal use is limited by widely variant individual factors, including height, weight, age, medical history, and the CYP3A4 and CYP2D6 enzymes and so on [18]. Before achieving the quetiapine maintenance dose, these influencing factors make it challenging to reach the narrow therapeutic window, which is monitored by the therapeutic drug monitoring from AGNP and a sub- or supra-therapeutic recommended therapeutic reference range (200–750 ng/ml). This may render treatment ineffective or increase the risk of sedation, hypotension, dry mouth, constipation, and tachycardia. Therefore, it is critical to help clinicians select the appropriate quetiapine dose and individualized quetiapine treatment using prediction models.

Recently, there has been a trend toward using machine learning and deep learning methods to create customized medications based on research from real-world situations [19]. With the help of large-scale complex algorithms and datasets, machine learning and deep learning algorithms, a branch of artificial intelligence, are able to predict clinical outcomes with high accuracy [20, 21]. When predicting from a variety of variables, they can assess data-driven estimation and derive nonlinear variable linkages [20, 21]. Several studies have utilized machine learning and deep learning approaches to improve the model depiction of the complex link between individual characteristics and drug dose, such as a vancomycin treatment prediction system using Extreme Gradient Boosting (XGBoost) [22], and a brand-new warfarin maintaining dose prediction system using Light Gradient Boosting Machine (LightGBM) [23].

Herein, our goal was to build a prediction model of quetiapine adjusted dose in a stationary state using algorithms based on machine learning and deep learning to support clinical prescription decisions. We did this by maximizing the use of real-world data to find significant influencing variables for quetiapine dose.

## Methods

### Patients and data

We included 474 patients with depression, who were treated with quetiapine and hospitalized in the First Hospital of Hebei Medical University, from November 1, 2019, to August 31, 2022.

**Fig. 1:**
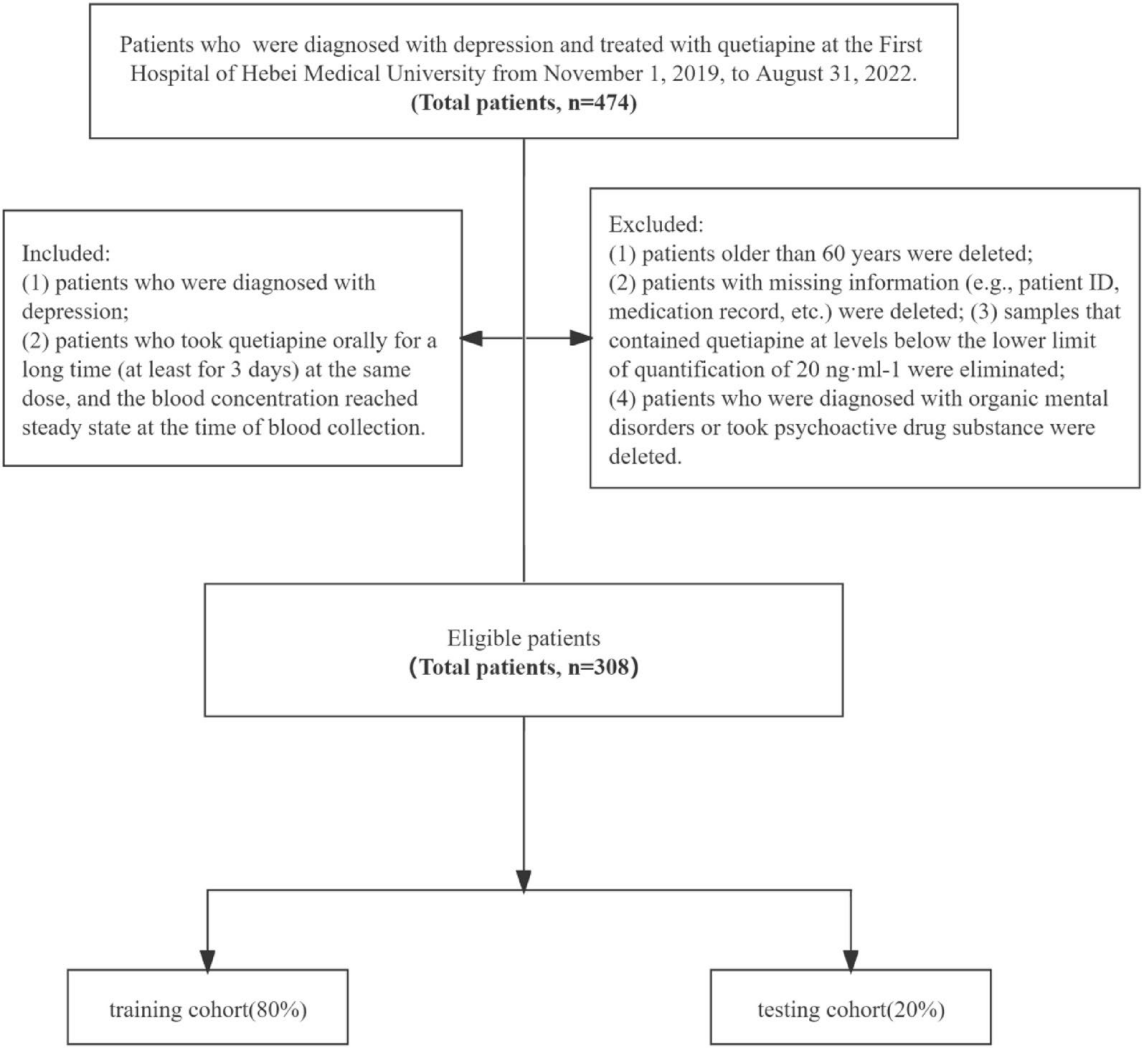
Workflow of sample selection

The inclusion criteria included the following: (1) patients who were diagnosed with depression and (2) patients who took quetiapine orally for a long time (at least for 3 days) at the same dose, and the blood concentration reached steady state at the time of blood collection. The exclusion criteria were as following: (1) patients older than 60 years were deleted; (2) patients with missing information (e.g., patient ID, medication record, etc.) were deleted; (3) samples that contained quetiapine at levels below the lower limit of quantification of 20 ng·ml^−1^ were eliminated; and (4) patients who were diagnosed with organic mental disorders or took psychoactive drug substance were deleted. The International Classification of diseases-10 (ICD-10) was used for diagnosis, and the supervising doctor made a diagnosis of depression. According to the Chinese Guidelines for the Diagnosis and Treatment of Mental Disorders 2020 Edition, antidepressants should be used as single as possible for patients with depression. When changing medicine is ineffective, combination therapy may be considered. The combination of two antidepressants with different mechanisms of action can be used, and other combinations include the combination of second-generation antipsychotics and lithium [24]. The First Hospital of Hebei Medical University is a tertiary hospital in local, and most of the patients who admitted in our hospital had poor effect after single antidepressant treatment and changing medicines in primary hospitals. Therefore, depending on the patient’s condition and guidelines, antidepressants combined with second generation antipsychotics (such as quetiapine) were commonly prescribed. Herein, the primary purpose of using quetiapine is the synergistic treatment of depression. All data were gathered from clinical paper records and computerized medical records held by the hospital for patients. Eventually, 308 eligible individuals were enrolled in this study. Figure 1 provides an illustration of the sample selection workflow.

### Data collection and processing

**Fig. 2:**
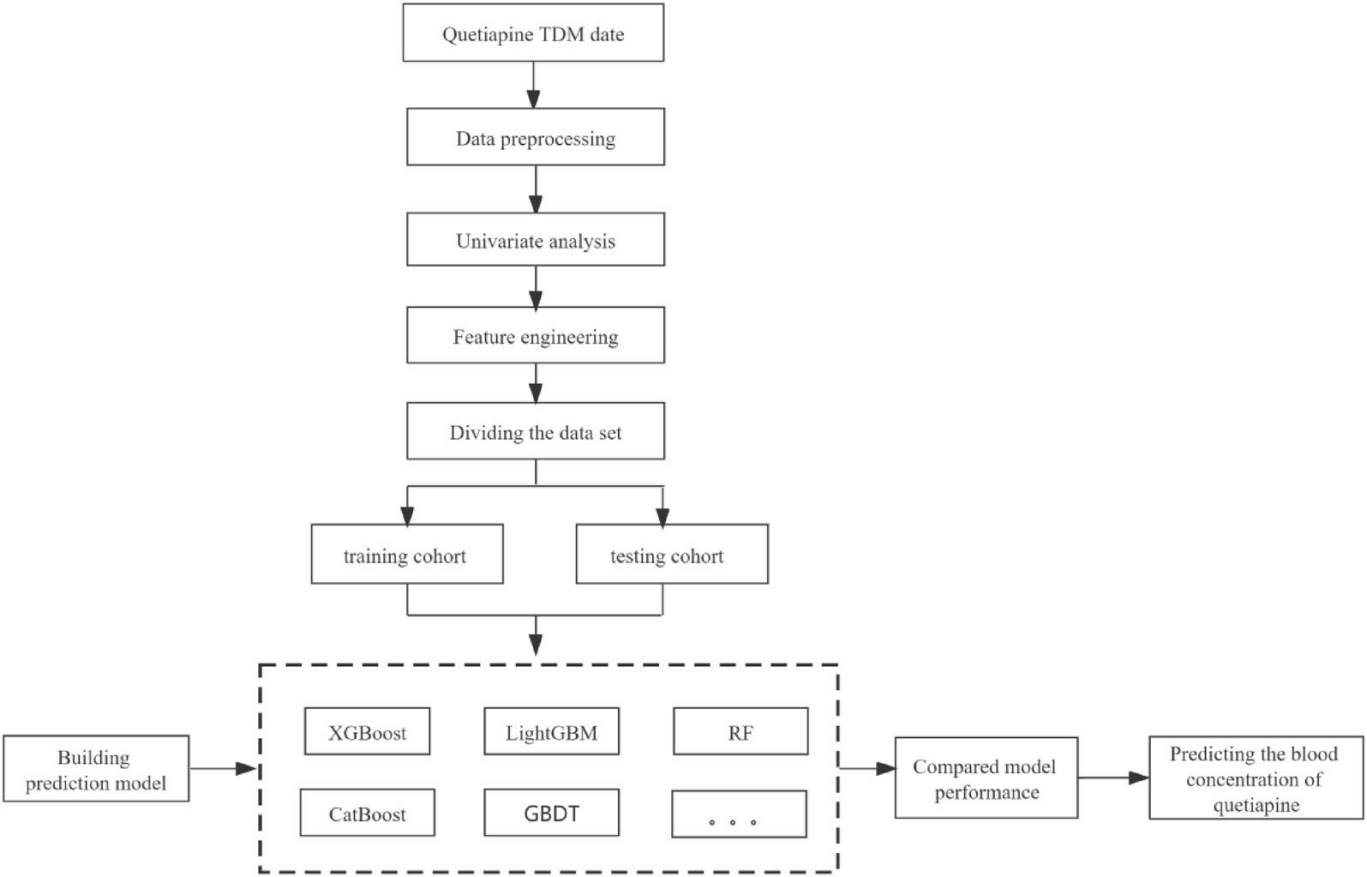
Process for establishing models and analyzing data

Figure 2 provides an illustration of data collecting and processing. First, based on the database's available data, we collected 47 clinical variables, including quetiapine administration information (e.g., daily dose and concentration), demographic information (e.g., age, gender, weight, height), comorbidities (e.g., hypertension, diabetes, hyperlipidemia), combination medication (e.g., CYP3A4 enzyme inhibitors, and CYP2D6/CYP3A4 competitive substrates) and laboratory parameters (e.g., regular blood test, liver function, and renal function). Considering the missing rates or extremely unbalanced variables, we preprocessed the obtained data, and the variables' missing values were filled with the mean.

### Variable selection and model establishment

As depicted in Fig. 2, univariate analysis was used to screen the variables after data collection from all relevant samples. Ultimately, variables which had p < 0.05 were selected. Based on the final dataset, the whole dataset was randomly divided into training cohort and testing cohort at the ratio of 80%: 20%. The data of the training cohort is used to train the model, and the test cohort is used to verify the final effect of the model. In this study, 246 subjects were in the training cohort and 62 subjects were in the testing cohort. Following that, key variables with p < 0.05 were chosen, and the daily dose of quetiapine was defined as the target variable. We created and evaluated nine different machine learning and deep learning models to compare the prediction abilities, including XGBoost, LightGBM, Random Forest (RF), Gradient Boosting (GBDT), Artificial Neural Network (ANN), Lasso Regression (LR), Support Vector Machine (SVM), TabNet and Decision Tree (DT). Assessment indicators were used for model evaluation, including precision, recall, F1-score, accuracy, sensitivity, and specificity. At the same time, we evaluated the effectiveness (AUROC) of quetiapine at various doses (100 mg/d, 200 mg/d, 300 mg/d, and 400 mg/d). Among these evaluation indicators, precision denotes the proportion of false positives [25]. Recall/sensitivity measures false negatives against true positives [25]. The F1-score is the harmonic average of the precision and recall [25]. Specificity measures false positives against true negatives [25]. The area under the ROC curve, or AUROC, is a comprehensive measurement that reflects the sensitivity and specificity of continuous variables. Accuracy is the proportion of correct predictions over the output results [25]. By contrasting the models' overall average accuracy, we may assess how well these models perform in terms of classification. After that, the confusion matrix, a special table used to view a classification model's performance, was then used to evaluate the prediction findings [26]. To avoid model overfitting and reduce bias, we used grid search combined with tenfold cross validation for hyperparameter tuning. Parameters of all nine models are displayed in Additional file 2: Table S1.

### Statistical analysis

IBM SPSS version 26.0 was used for statistical research. (IBM Corporation, Armonk, New York, USA). In the comparison between training cohort and testing cohort, Mann–Whitney *U* test (non-normal distribution) and independent *t* test (normal distribution) were used to analyze the various continuous factors. Categorical data were analyzed by the Chi-squared test (n ≥ 5) or Fisher's exact test (n < 5). Statistical significance was set at *p* value < 0.05. Windows Python 3.9.12 was used to create each machine learning model.

## Results

### Baseline information

Table 1 displays the distribution of features across the complete dataset. This research included 308 depressed patients in total, 131 of whom were men and 177 of whom were women. Median (interquartile range, IQR) was used to characterize continuous variables, and frequency (percentage, %) was used to describe categorical variables. Patients’ average age was 19.00 (IQR 15.00–36.25) years. The median height and weight were 166.00 (IQR 160.00–172.00) cm and 65.00 (IQR 55.00–76.00) kg. Based on their daily quetiapine dose, the patients were separated into several groups, with 57 (18.51%) receiving a dose of 100 mg, 108 (35.06) receiving a dose of 200 mg, 74 (24.03%) receiving a dose of 300 mg, and 69 (22.03%) receiving a dose of 400 mg. The median value of the serum levels in the dataset was 213.06 (IQR 124.23–370.24) ng·ml^−1^. Comorbidities including hypertension, diabetes, and hyperlipidemia occupied 8.44%, 10.00%, and 7.14%, respectively. Combination medicine usage rates for CYP3A4 enzyme inhibitors were 0.32%, CYP3A4 competitive substrates were 5.19%, and CYP2D6 competitive substrates were 9.74%.

**Table 1:** Description of the study samples

Category	Variable	Median (IQR) | n(%)	Miss rate
Quetiapine information	TDM value, ng·ml^−1^, median (IQR)	213.06 (124.23–370.24)	0.00%
	Daily dose, median (IQR)		0.00%
	100 (1)	57 (18.51%)	
	200 (2)	108 (35.06%)	
	300 (3)	74 (24.03%)	
	400 (4)	69 (22.40%)	
Demographic information	Age, median (IQR)	19.00 (15.00–36.25)	0.00%
	Sex, n (%)		0.00%
	Female	177 (57.47%)	
	Male	131 (42.53%)	
	Weight, kg, median (IQR)	65.00 (55.00–76.00)	52.92%
	Height, cm, median (IQR)	166.00 (160.00–172.00)	53.25%
Concomitant diseases	Hypertension, n (%)	26 (8.44%)	0.00%
	Diabetes, n (%)	10 (3.25%)	0.00%
	Hyperlipidemia, n (%)	22 (7.14%)	0.00%
Combination medication	CYP3A4 enzyme inhibitors, n (%)	1 (0.32%)	0.00%
	CYP3A4 competitive substrates, n (%)	16 (5.19%)	0.00%
	CYP2D6 competitive substrates, n (%)	30 (9.74%)	0.00%
Laboratory parameters	AFU, U/L, median (IQR)	17.20 (14.05–20.20)	9.42%
	α-HBDH, U/L, median (IQR)	100.00 (89.05–114.00)	24.03%
	GGT, U/L, median (IQR)	17.00 (13.00–28.00)	3.57%
	ALT, U/L, median (IQR)	16.20 (10.30–29.10)	3.57%
	NEUR, %, median (IQR)	51.25 (45.85–59.35)	5.19%
	LDH, U/L, median (IQR)	158.00 (142.25–178.00)	24.03%
	MONOR, %, median (IQR)	8.05 (6.50–9.40)	5.19%
	BASOR, %, median (IQR)	0.40 (0.30–0.60)	10.06%
	EOSR, %, median (IQR)	2.50 (1.70–3.60)	10.06%
	AST, U/L, median (IQR)	18.50 (15.40–22.90)	3.57%
	BUN, mmol·L^−1^, median (IQR)	3.96 (3.15–4.57)	3.57%
	UA, µmol·L^−1^, median (IQR)	332.45 (276.80–401.45)	3.25%
	MCV, fl, median (IQR)	88.60 (85.55–92.03)	5.19%
	MPV, fl, median (IQR)	8.70 (8.00–9.50)	5.19%
	MCHC, g·L^−1^, median (IQR)	338.00 (331.00–345.00)	5.19%
	MCH, pg, median (IQR)	30.20 (28.80–31.20)	5.19%
	TBA, µmol·L^−1^, median (IQR)	3.00 (1.98–5.00)	1.30%
	TBIL, µmol·L^−1^, median (IQR)	8.50 (6.80–10.93)	1.30%
	TP, g·L^−1^, median (IQR)	65.10 (63.00–68.95)	1.30%
	LYP, %, median (IQR)	35.25 (28.55–40.75)	5.19%
	LYM, 10^9^/L, median (IQR)	2.00 (1.64–2.40)	5.19%
	GLB, g·L^−1^, median (IQR)	24.40 (22.40–26.80)	1.30%
	AIB/GLB, median (IQR)	1.68 (1.52–1.85)	1.30%
	WBC, 10^9^/L, median (IQR)	5.90 (4.97–6.80)	5.19%
	AIB, g·L^−1^, median (IQR)	40.70 (39.00–43.00)	1.30%
	DBIL, µmol·L^−1^, median (IQR)	1.70 (1.30–2.30)	1.30%
	HCT, %, median (IQR)	37.80 (34.90–41.70)	5.19%
	RBC, 10^12^/L, median (IQR)	4.34 (4.03–4.70)	5.19%
	Cr, µmol·L^−1^, median (IQR)	60.30 (53.50–72.70)	3.57%
	CK, U/L, median (IQR)	71.00 (52.00–101.75)	24.03%
	ChE, U/L, median (IQR)	7524.00 (6640.75–8536.00)	1.30%
	ADA, U/L, median (IQR)	9.50 (7.90–11.65)	9.42%
	PLT, 10^9^/L, median (IQR)	238.00 (199.75–277.25)	5.19%
	Hb, g·L^−1^, median (IQR)	130.00 (118.00–142.00)	5.19%
	IBIL, µmol·L^−1^, median (IQR)	6.90 (5.40–8.90)	1.30%

*AFU* a-L-fucosidase, *HBDH* alpha-hydroxybutyrate dehydrogenase, *GGT* γ-glutamyl transpeptidase, *ALT* alanine aminotransferase, *NEUR* percentage of neutrophils, *LDH* lactic dehydrogenase, *MONOR* percentage of monocytes, *BASOR* percentage of basophilic granulocyte, *EOSR* percentage of eosinophils granulocyte, *AST* aspartate aminotransferase, *ALT* alanine aminotransferase, *BUN* blood urea nitrogen, *UA* uric acid, *MCV* mean corpuscular volume, *MPV* mean platelet volume, *MCHC* mean corpuscular hemoglobin concentration, *MCH* mean corpuscular hemoglobin, *TBA* total bile acid, *TBIL* total bilirubin, *TP* total protein, *LYP* percentage of lymphocytes, *GLB* globulin, *WBC* white blood cells, *AIB* albumin, *DBIL* direct bilirubin, *RDW* Red cell distribution width, *HCT* hematocrit, *RBC* red blood cell count, *Cr* creatinine, *CK* creatine kinase, *ChE* cholinesterase, *ADA* adenosine deaminase, *Hb* hemoglobin, *IBIL* Indirect Bilirubin

### Variable analysis

Considering extremely unbalanced variables, including hypertension, diabetes, hyperlipidemia, CYP3A4 enzyme inhibitors/inducers/competitive substrates, CYP2D6 enzyme inhibitors/competitive substrates, and variables with a missing rate greater than 50%, including weight, height may influence the predicted results of quetiapine, we preprocessed the obtained data before determining the significant associations between univariates. This led to a total of 38 candidate predictors, and finally four variables were selected which had p < 0.05, including quetiapine TDM value, age, mean corpuscular hemoglobin concentration (MCHC), and total bile acid (TBA), described in Table 2.

**Table 2:** Outcomes of the univariate analysis

Variable	Statistic	p value
SEX	0.212	0.976
TDM VALUE	139.612	< 0.001
AGE	32.840	< 0.001
AFU	3.116	0.374
α-HBDH	2.329	0.507
GGT	3.155	0.368
ALT	5.561	0.135
NEUR	1.287	0.732
LDH	2.695	0.441
MONOR	1.740	0.628
BASOR	3.328	0.344
EOSR	0.466	0.926
AST	1.046	0.790
BUN	3.491	0.322
UA	5.899	0.117
MCV	3.846	0.279
MPV	3.921	0.270
MCHC	7.699	0.053
MCH	6.508	0.089
TBA	8.216	0.042
TBIL	3.471	0.325
TP	2.146	0.543
LYP	1.672	0.643
LYM	2.338	0.505
GLB	4.594	0.204
AIB/GLB	5.506	0.138
WBC	1.114	0.774
AIB	3.067	0.381
DBIL	2.436	0.487
HCT	0.854	0.837
RBC	0.232	0.972
Cr	5.646	0.130
CK	1.766	0.622
ChE	2.736	0.434
ADA	5.345	0.148
PLT	1.564	0.667
Hb	1.242	0.743
IBIL	4.974	0.174

*AFU* a-L-fucosidase, *HBDH* alpha-hydroxybutyrate dehydrogenase, *GGT* γ-glutamyl transpeptidase, *ALT* alanine aminotransferase, *NEUR* percentage of neutrophils, *LDH* lactic dehydrogenase, *MONOR* percentage of monocytes, *BASOR* percentage of basophilic granulocyte, *EOSR* percentage of eosinophils granulocyte, *AST* aspartate aminotransferase, *ALT* alanine aminotransferase, *BUN* blood urea nitrogen, *UA* uric acid, *MCV* mean corpuscular volume, *MPV* mean platelet volume, *MCHC* mean corpuscular hemoglobin concentration, *MCH* mean corpuscular hemoglobin, *TBA* total bile acid, *TBIL* total bilirubin, *TP* total protein, *LYP* percentage of lymphocytes, *GLB* globulin, *WBC* white blood cells, *AIB* albumin, *DBIL* direct bilirubin, *RDW* Red cell distribution width, *HCT* hematocrit, *RBC* red blood cell count, *Cr* creatinine, *CK* creatine kinase, *ChE* cholinesterase, *ADA* adenosine deaminase, *Hb* hemoglobin, *IBIL* Indirect Bilirubin

### Model establishment and validation

We developed and validated prediction models based on the selected features using nine algorithms (including XGBoost, LightGBM, RF, GBDT, SVM, LR, ANN, TabNet, and DT). Table 3 displays the performance of these models in testing cohort. The metrics of the XGBoost model outperformed those of other models and achieved the best overall performance, with precision = 0.91 ± 0.07, recall = 0.68 ± 0.1, F1 score = 0.78 ± 0.09, AUROC = 0.93 ± 0.04, sensitivity = 0.68 ± 0.1, and specificity = 0.98 ± 0.01 for predicting the daily dose of 100 mg quetiapine; precision = 0.67 ± 0.05, recall = 0.76 ± 0.09, F1 score = 0.71 ± 0.03, AUROC = 0.77 ± 0.04, sensitivity = 0.76 ± 0.09, and specificity = 0.78 ± 0.05 for predicting the daily dose of 200 mg quetiapine; precision = 0.67 ± 0.17, recall = 0.47 ± 0.09, F1 score = 0.54 ± 0.1, AUROC = 0.77 ± 0.08, sensitivity = 0.47 ± 0.09, and specificity = 0.93 ± 0.04 for predicting the daily dose of 300 mg quetiapine; precision = 0.64 ± 0.1, recall = 0.79 ± 0.08, F1 score = 0.7 ± 0.07, AUROC = 0.86 ± 0.06, sensitivity = 0.79 ± 0.08, and specificity = 0.86 ± 0.05 for predicting the daily dose of 400 mg quetiapine, and accuracy = 0.69 ± 0.03 for the entire XGBoost model. As a result, XGBoost was chosen to forecast the daily dose of quetiapine.

**Table 3:** Nine different algorithms' model performance metrics

Model	label	precision	recall	f1-score	support	AUROC	sensitivity	specificity	accuracy
XGBoost									0.69 ± 0.03
	0	0.91 ± 0.07	0.68 ± 0.1	0.78 ± 0.09	11.4 ± 1.71	0.93 ± 0.04	0.68 ± 0.1	0.98 ± 0.01	
	1	0.67 ± 0.05	0.76 ± 0.09	0.71 ± 0.03	22.8 ± 2.25	0.77 ± 0.04	0.76 ± 0.09	0.78 ± 0.05	
	2	0.67 ± 0.17	0.47 ± 0.09	0.54 ± 0.1	13.3 ± 2.58	0.77 ± 0.08	0.47 ± 0.09	0.93 ± 0.04	
	3	0.64 ± 0.1	0.79 ± 0.08	0.7 ± 0.07	14.5 ± 2.32	0.86 ± 0.06	0.79 ± 0.08	0.86 ± 0.05	
LGBM									0.6 ± 0.05
	0	0.86 ± 0.1	0.75 ± 0.1	0.8 ± 0.09	11.4 ± 1.71	0.93 ± 0.05	0.75 ± 0.1	0.97 ± 0.02	
	1	0.59 ± 0.07	0.62 ± 0.1	0.6 ± 0.06	22.8 ± 2.25	0.75 ± 0.03	0.62 ± 0.1	0.75 ± 0.05	
	2	0.45 ± 0.18	0.41 ± 0.14	0.42 ± 0.14	13.3 ± 2.58	0.72 ± 0.1	0.41 ± 0.14	0.87 ± 0.05	
	3	0.59 ± 0.11	0.63 ± 0.07	0.61 ± 0.07	14.5 ± 2.32	0.85 ± 0.05	0.63 ± 0.07	0.86 ± 0.06	
RF									0.55 ± 0.05
	0	0.81 ± 0.06	0.75 ± 0.12	0.77 ± 0.08	11.4 ± 1.71	0.92 ± 0.05	0.75 ± 0.12	0.96 ± 0.02	
	1	0.54 ± 0.07	0.54 ± 0.1	0.54 ± 0.06	22.8 ± 2.25	0.7 ± 0.04	0.54 ± 0.1	0.73 ± 0.05	
	2	0.42 ± 0.13	0.47 ± 0.14	0.43 ± 0.1	13.3 ± 2.58	0.73 ± 0.09	0.47 ± 0.14	0.82 ± 0.05	
	3	0.53 ± 0.14	0.5 ± 0.12	0.51 ± 0.11	14.5 ± 2.32	0.81 ± 0.05	0.5 ± 0.12	0.86 ± 0.05	
GBDT									0.54 ± 0.05
	0	0.77 ± 0.11	0.73 ± 0.13	0.75 ± 0.1	11.4 ± 1.71	0.92 ± 0.04	0.73 ± 0.13	0.95 ± 0.03	
	1	0.56 ± 0.1	0.54 ± 0.09	0.54 ± 0.06	22.8 ± 2.25	0.7 ± 0.04	0.54 ± 0.09	0.75 ± 0.06	
	2	0.34 ± 0.13	0.39 ± 0.16	0.36 ± 0.13	13.3 ± 2.58	0.68 ± 0.1	0.39 ± 0.16	0.8 ± 0.05	
	3	0.56 ± 0.14	0.54 ± 0.12	0.54 ± 0.1	14.5 ± 2.32	0.8 ± 0.05	0.54 ± 0.12	0.87 ± 0.05	
SVM									0.64 ± 0.06
	0	0.94 ± 0.07	0.78 ± 0.11	0.85 ± 0.07	11.4 ± 1.71	0.94 ± 0.05	0.78 ± 0.11	0.99 ± 0.01	
	1	0.63 ± 0.08	0.8 ± 0.09	0.7 ± 0.06	22.8 ± 2.25	0.79 ± 0.03	0.8 ± 0.09	0.72 ± 0.07	
	2	0.52 ± 0.19	0.2 ± 0.1	0.26 ± 0.09	13.3 ± 2.58	0.74 ± 0.08	0.2 ± 0.1	0.95 ± 0.03	
	3	0.57 ± 0.14	0.69 ± 0.08	0.62 ± 0.1	14.5 ± 2.32	0.85 ± 0.06	0.69 ± 0.08	0.83 ± 0.08	
LR									0.65 ± 0.07
	0	0.88 ± 0.11	0.74 ± 0.12	0.8 ± 0.11	11.4 ± 1.71	0.94 ± 0.04	0.74 ± 0.12	0.98 ± 0.02	
	1	0.63 ± 0.08	0.77 ± 0.11	0.69 ± 0.07	22.8 ± 2.25	0.8 ± 0.03	0.77 ± 0.11	0.74 ± 0.06	
	2	0.52 ± 0.15	0.3 ± 0.17	0.36 ± 0.11	13.3 ± 2.58	0.76 ± 0.06	0.3 ± 0.17	0.92 ± 0.05	
	3	0.62 ± 0.13	0.71 ± 0.1	0.66 ± 0.1	14.5 ± 2.32	0.86 ± 0.06	0.71 ± 0.1	0.86 ± 0.06	
ANN									0.59 ± 0.04
	0	0.94 ± 0.08	0.63 ± 0.13	0.74 ± 0.09	11.4 ± 1.71	0.94 ± 0.04	0.63 ± 0.13	0.99 ± 0.01	
	1	0.55 ± 0.06	0.82 ± 0.12	0.65 ± 0.03	22.8 ± 2.25	0.73 ± 0.08	0.82 ± 0.12	0.61 ± 0.1	
	2	0.51 ± 0.27	0.21 ± 0.17	0.26 ± 0.14	13.3 ± 2.58	0.74 ± 0.06	0.21 ± 0.17	0.95 ± 0.05	
	3	0.6 ± 0.15	0.62 ± 0.08	0.6 ± 0.07	14.5 ± 2.32	0.83 ± 0.05	0.62 ± 0.08	0.86 ± 0.08	
TabNet									0.51 ± 0.06
	0	0.59 ± 0.28	0.49 ± 0.23	0.51 ± 0.21	11.4 ± 1.71	0.82 ± 0.06	0.49 ± 0.23	0.93 ± 0.07	
	1	0.57 ± 0.1	0.54 ± 0.13	0.54 ± 0.1	22.8 ± 2.25	0.68 ± 0.06	0.54 ± 0.13	0.75 ± 0.13	
	2	0.31 ± 0.16	0.28 ± 0.15	0.28 ± 0.14	13.3 ± 2.58	0.61 ± 0.13	0.28 ± 0.15	0.84 ± 0.08	
	3	0.52 ± 0.13	0.67 ± 0.13	0.58 ± 0.1	14.5 ± 2.32	0.8 ± 0.07	0.67 ± 0.13	0.8 ± 0.08	
DT									0.56 ± 0.07
	0	0.73 ± 0.16	0.66 ± 0.16	0.68 ± 0.12	11.4 ± 1.71	0.86 ± 0.06	0.66 ± 0.16	0.94 ± 0.04	
	1	0.56 ± 0.1	0.65 ± 0.11	0.59 ± 0.07	22.8 ± 2.25	0.72 ± 0.08	0.65 ± 0.11	0.69 ± 0.12	
	2	0.38 ± 0.15	0.28 ± 0.18	0.3 ± 0.15	13.3 ± 2.58	0.66 ± 0.08	0.28 ± 0.18	0.89 ± 0.07	
	3	0.58 ± 0.11	0.61 ± 0.17	0.59 ± 0.13	14.5 ± 2.32	0.81 ± 0.06	0.61 ± 0.17	0.86 ± 0.07	

Label 0 indicates patients with daily dose of 100 mg, Label 1 indicates patients with daily dose of 200 mg, Label 2 indicates patients with daily dose of 300 mg, and Label 3 indicates patients with daily dose of 400 mg

On this basis, XGBoost calculated and ranked the importance scores of four selected variables, as shown in Table 4. Among them, the most important feature in the prediction model was discovered to be the quetiapine TDM value (importance score = 0.41 ± 0.02), followed by AGE (importance score = 0.23 ± 0.01), MCHC (importance score = 0.19 ± 0.01) and TBA (importance score = 0.18 ± 0.01).

**Table 4:** Importance score ranking of variables by XGBoost

Variable	Importance
Quetiapine TDM value	0.41 ± 0.02
AGE	0.23 ± 0.01
MCHC	0.19 ± 0.01
TBA	0.18 ± 0.01

**Fig. 3:**
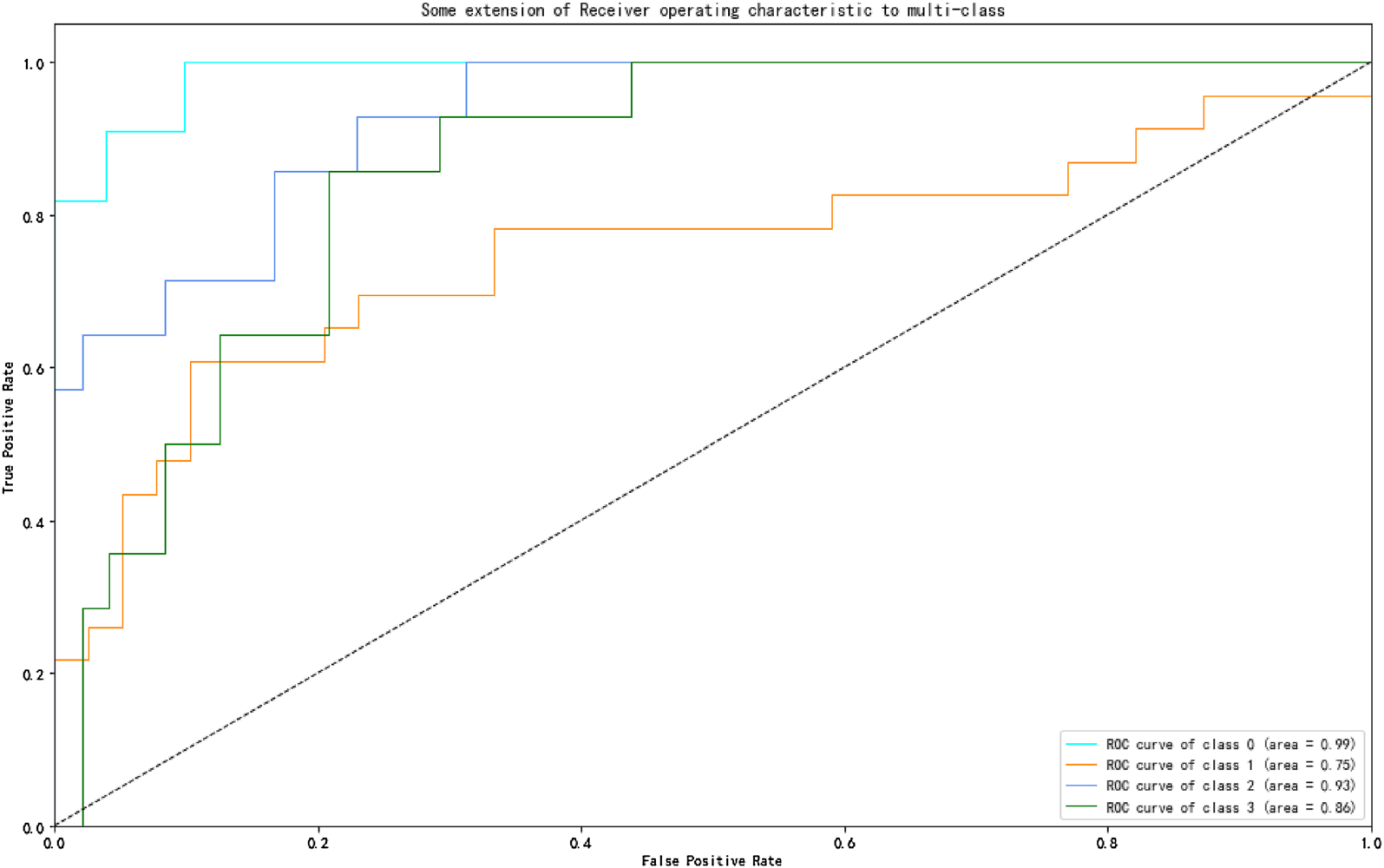
ROC curve at different doses. Class 0 indicates patients with daily dose of 100 mg, Class 1 indicates patients with daily dose of 200 mg, Class 2 indicates patients with daily dose of 300 mg, and Class 3 indicates patients with daily dose of 400 mg

Then, we evaluated the performance of XGBoost model with 4 variables (quetiapine TDM value, AGE, MCHC, and TBA) using a testing cohort of 62 patients. Figure 3 shows the AUROC values for XGBoost under different groups according to the daily dose of quetiapine. Typically, an AUROC has a value between 0.5 and 1.0, and the larger AUROC indicates the greater model classification effect. Based on different dose intervals, the patients were separated into four subgroups: those with a daily dose of 100 mg (11 cases), 200 mg (23 cases), 300 mg (14 cases), and 400 mg (14 cases). In different subgroups according to the quetiapine daily dose of 100 mg, 200 mg, 300 mg and 400 mg, AUROC were 0.99, 0.75, 0.93, and 0.86, respectively.

**Fig. 4:**
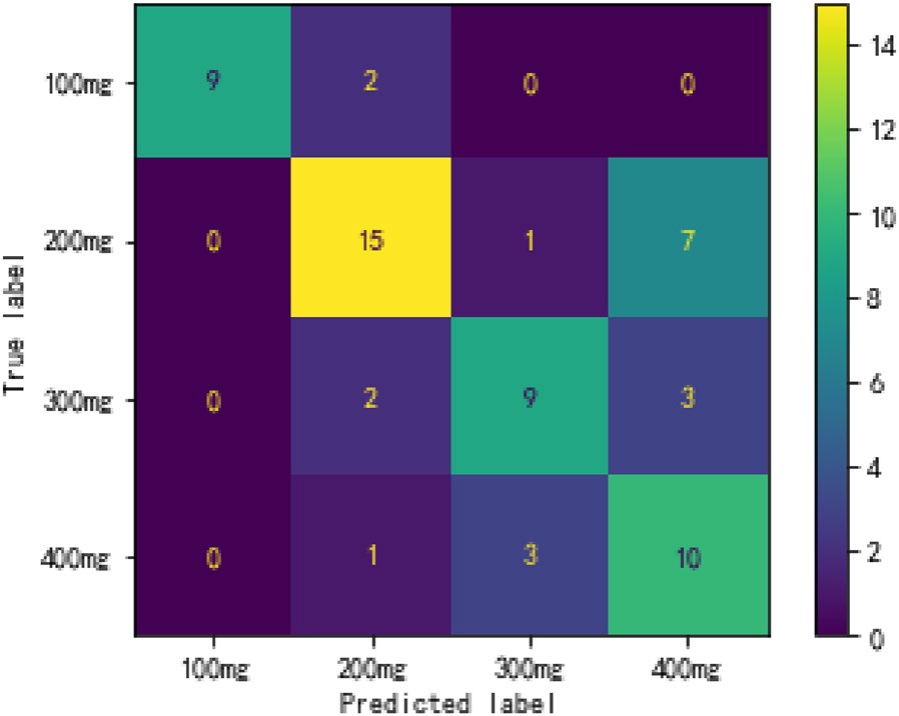
Confusion matrix in the CatBoost model

Figure 4 summarizes the model’s performance in the testing cohort (62 cases) through confusion matrix. The model accurately predicted the dose regimen of 100 mg, 200 mg, 300 mg, and 400 mg quetiapine for 9, 15, 9, and 10 individuals, respectively. The evaluation indicators of four subgroups in the XGBoost model were calculated. The model can predict the dose regimen of 100 mg quetiapin with 100% precision and 82% recall rate; the dose regimen of 200 mg with 75% precision and 65% recall rate; the dose regimen of 300 mg with 69% precision and 64% recall rate; and the dose regimen of 400 mg with 50% precision and 71% recall rate, respectively. The results showed that the predicted quetiapine dose metrics agreed well with those from the clinically delivered plans for these patients.

## Discussion

One of the most popular and efficient ways to treat depression in a therapeutic context is with antidepressants, which also has the ability to successfully slow the onset of disease in depressed patients. However, research indicates that only about half of major depressive disorder (MDD) patients receive antidepressants that work well for them, and only about a third of them experience remission [27]. The use of AAs as first-line medicines, notably quetiapine, is recommended by various current pharmacological augmentation guidelines for treating depression [17, 28, 29]. The right quetiapine dose and personalized quetiapine treatment are frequently challenging for clinicians.

To better estimate quetiapine dose during depression treatment and to find valid and accurate predictors, we compared the prediction abilities of quetiapine dose by applying nine machine learning and deep learning techniques for patients with depression. Ultimately, the XGBoost algorithm with the best performance (accuracy = 0.69) among nine models was selected to build the prediction model. Afterward, it can be observed that a number of 43 instances of quetiapine dose were properly predicted in the testing cohort. The overall accuracy of the model was 0.69. The moderate accuracy demonstrates that the effect of accurately predicting quetiapine dose is acceptable, and our findings may offer clinicians recommendations for prompt drug regimen adjustments. In addition, we performed dose subgroup analyses to show individual predictive performance across dose levels and to help refine model performance with continued recruitment of data for a given range of daily doses.

Calculations of the area under the concentration–time curve (AUC), for instance, provide the basis of classic pharmacokinetic studies. However, if the data are insufficient or cannot support a pharmacokinetic modeling technique, the model is erroneous [30]. Recently, it has been noticed that there is growing interest in novel statistical techniques, such as population pharmacokinetic (popPK) analysis. Nonlinear mixed-effects modeling (NONMEM) is the most popular method for this type of pharmacokinetic data analysis [31, 32]. Nevertheless, the PPK model is relatively inflexible to apply because of the explicit mathematical model used, and adding or removing a parameter may be challenging [33]. Machine learning, in contrast, is renowned for its self-organizational and learning skills, which let computers learn from “experience” without being explicitly taught [34, 35]. It is a form of artificial intelligence that enables systems to examine a wide range of data gathered from electronic health records (EHRs) and automatically learn from them using cutting-edge statistical and probabilistic techniques to make more precise predictions by building clever and efficient predictive models [36]. Recent years have seen a significant increase in study interest in the use of machine learning for clinical drug therapies, which leads to an increasingly significant impact on the development of personalized dosing, particularly in the choice of drug dose [37]. A few studies on the use of machine learning to forecast drug doses or blood concentrations have been reported [38–43].

In this study, we innovatively used machine learning and deep learning techniques to predict quetiapine dose based on real-world data. Machine learning models can be updated by automatically extracting EHR data and continuously monitoring physiological data, and are effective approaches to modeling real-world data. The commonly used PPK models have some limitations, such as difficulty in modeling, less consideration of influencing factors, and low accuracy. Herein, multi-level data mining was conducted by machine learning to screen out a variety of real-world influencing factors, to construct a more practical and accurate dose prediction model. Therefore, the combination of machine learning and dose prediction can help to improve the level of precision medicine in clinical.

We considered multiple algorithms for model establishment. DT is simple and easy to understand, but there is a risk of overfitting. RF uses bagging sampling, random attribute selection and model ensemble to address excessive risk decision tree learning. On the basis of RF, GBDT combined with Boosting establishes the connections between trees, making the forest an ordered collective decision-making system. XGBoost goes a step further than GBDT by adding regular terms to the objective function at each iteration to reduce the risk of overfitting, and it can integrate multiple decision trees to achieve the goal of regression or classification [44]. For models such as ANN and XGBoost, they perform quite well on large-scale datasets. However, good prediction results can also be obtained on small data sets by adjusting hyperparameters to avoid overfitting. Each algorithm has its advantages and disadvantages, the performance of different algorithms depends on the characteristics of the dataset, and the final selection of the algorithm is based on the computational results. Herein, we used grid search combined with tenfold cross validation to find the optimal hyperparameters and avoid overfitting to obtain the optimal model.

The significant predictor for predicting quetiapine dose is the quetiapine concentration. Several studies on psychotic disorders have identified that dose affects quetiapine concentration. According to a review, quetiapine had linear pharmacokinetics in the studied dose range, and had predictable pharmacokinetics [45]. Albantakis et al. have also quantified the relationship between daily dose and serum concentration in children and adolescents with psychotic and mood disorders. Between the daily dose and quetiapine serum levels (from trough samples) in the entire sample, they discovered a statistically significant, positive, but flimsy linear connection [46]. Among the crucial parameters we chose for our study's prediction model, the concentration was the most prominent influencing variable, and it was positively associated with quetiapine dose, which was in line with earlier research.

The effect of age on the metabolism of second-generation antipsychotics has been described in a few prior investigations. One study revealed that dose-adjusted concentrations of quetiapine increased by an average of 13% per decade from the age of 20 [47], while another found that the average concentrations were 67% higher in patients over the age of 70 compared to those between the ages of 18 and 69 [48]. Another study found that patients aged 65 and above had 50% higher plasma concentrations than younger patients [49]. For children and adolescents (10–17 years of age), at steady state, the pharmacokinetics of the parent compound were similar to adults. However, when adjusted for dose and weight, AUC and Cmax of the parent compound were 41% and 39% lower, respectively, in children and adolescents than in adults [50, 51]. In our study, patients older than 60 years were excluded because of the small number of senior patients that model can only learn little information. The ability of the elderly to metabolize and excrete drugs may be reduced, which may lead to the accumulation of drugs in the body, and liver and kidney function may also be affected. As a result, older people tend to require smaller doses of drugs. In this study, age is one of the most important feature in the final prediction model. In the following study, we will include more patients older than 60 years in the model to verify its generalizability.

In addition, some previous studies have indicated that low MCHC increases the likelihood of developing pathological disorders, such as poor functional status, dementia, and cognitive decline as well as morbidity and death [50–53]. Poor functional status, such as decreased ability to carry oxygen, may lead to changes in the pharmacokinetics of quetiapine and thus affect the dose of quetiapine. Meanwhile, because it is a measure determined from the haemoglobin concentration (HGB) divided by mean cellular volume (MCV) and red blood cell count (RBC), the MCHC is a good indicator to detect anaemia [54]. Depending on the demographic data investigated, anemia, a condition marked by a deficiency in hemoglobin in the blood, affects an estimated percentage from 2.9% to 60.1% of older persons [55]. Many illnesses, including malnutrition, obesity, cancer, chronic renal disease, are linked to anemic people, which may lead to changes in the pharmacokinetics of quetiapine and thus affect its dose.

Furthermore, hepatic metabolism accounts for the majority of quetiapine elimination, and less than 1% of the amount taken orally after a single administration was excreted unaltered, showing quetiapine is rapidly metabolized [56, 57]. According to studies, people with liver disease (n = 8) had a 30% lower mean oral clearance of quetiapine than patients with normal liver function. Two of 8 patients with hepatic impairment experienced a threefold increase in AUC and Cmax compared with healthy patients [56, 57]. TBA is closely related to liver function and abnormally high value suggests poor liver health. Abnormal TBA levels indicate that patients may have impaired liver function, which may inhibit metabolism of quetiapine in the liver, resulting in high quetiapine concentration and dose adjustment may be needed. In one word, quetiapine TDM value, age, MCHC, and TBA, show important associations with quetiapine dose, which could be used as the predictors in the individualized medication model of quetiapine, to help clinicians choose the reasonable regimen.

In different dose groups, according to Additional file 1: Figure S1, the blood concentration points of some patients with a dose of 200 mg are extreme outliers, and there is a crossover with the upper quartile concentration points of patients with a dose of 400 mg. Also, there is a crossover between the upper quartile concentration points of patients with a dose of 200 mg and the lower quartile concentration points of patients with a dose of 300 mg. All the situations of crossover may affect the clinician’s regimen choice and the prediction outcome in 200 mg group. Therefore, the AUROC for 200 mg group is lower than other dose groups.

Our model has some notable flaws. First, due to the availability of data, such as extremely uneven distribution, lots of missing values and so on, some variables were excluded. A future goal is to improve the model when a great deal of samples may be used to thoroughly study the factors. Second, our model has not been sufficiently tested on additional data sets. By using the model on a larger pooled data set, future studies could delve deeper into these problems. The identification of more potent predictors and the improvement of prediction accuracy are likely to result from the input of additional data. Third, due to the constraints of the test conditions, several pertinent patient characteristics (such as CYP450 polymorphisms) were excluded. Last, in this study, some underlying confounding factors were not analyzed, such as the using duration of quetiapine, prior use of antipsychotics, mood stabilizers and antidepressants before admission, drug combination of benzodiazepines, anxiolytics, and lithium, and complex clinical situations including severity of illness and multiple complications [58]. There is a drawback of real-world study that there exist some unknown confounders from real clinical settings. In future study, we expect to apply propensity score matching and stratified analysis for reducing confounding bias.

According to our knowledge, this research is the initial to use XGBoost algorithm for estimating the dose of quetiapine for patients with depression. Our study could identify important influencing variables for quetiapine dose by maximizing the use of real-world data to support quetiapine dose adjustments for each patient. In clinical applications, we expect to develop a web tool for drug dose calculation that can automatically generate recommended quetiapine doses by entering the values of key variables (such as quetiapine TDM value, age, MCHC, and TBA) based on electronic medical records, blood tests and TDM, providing clinical decision support to improve therapeutic response and reduce patient’s burden.

## Conclusion

In this work, machine learning techniques are used for the first time to estimate the dose of quetiapine for patients with depression, which is important and valuable for the clinical drug recommendations. Our model was designed as a real-time assisting clinical decision support tool to balance the effect of quetiapine dose on both treatment efficacy and toxicity outcomes, and to maximize the benefit of treatment for each patient. Therefore, our study fills the gap in this research field.

### Supplementary Information

**Additional file 1.** **Figure S1.** Boxplot of different doses.

**Additional file 2.** **Table S1.** Parameters of nine models.

## Data Availability

All data generated and analyzed during this study are included in this published article.

## Abbreviations

XGBoost: Extreme Gradient Boosting
LightGBM: Light Gradient Boosting Machine
RF: Random Forest
GBDT: Gradient Boosting
SVM: Support vector machine
LR: Lasso Regression
ANN: Artificial neural network
TabNet: Attentive Interpretable Tabular Learning
DT: Decision tree
TDM: Therapeutic drug monitoring
AUROC: Area under the receiver operating characteristic
WHO: World Health Organization
SSRI: Serotonin reuptake inhibitors
SNRI: Serotonin–norepinephrine reuptake inhibitors
NaSSA: Noradrenergic and specific serotonergic antidepressants
CANMAT: Canadian network for mood and anxiety treatments
AA: Atypical antipsychotics
EMA: European Medicine Agency
XR: Extended-release
CYP: CytochromeP450
IQR: Interquartile range
AFU: A-L-fucosidase
HBDH: Alpha-hydroxybutyrate dehydrogenase
GGT: γ Glutamyl transpeptidase
ALT: Alanine aminotransferase
NEUR: Percentage of neutrophils
LDH: Lactic dehydrogenase
MONOR: Percentage of monocytes
BASOR: Percentage of basophilic granulocyte
EOSR: Percentage of eosinophils granulocyte
AST: Aspartate aminotransferase
ALT: Alanine aminotransferase
BUN: Blood urea nitrogen
UA: Uric acid
MCV: Mean corpuscular volume
MPV: Mean platelet volume
MCHC: Mean corpuscular hemoglobin concentration
MCH: Mean corpuscular hemoglobin
TBA: Total bile acid
TBIL: Total bilirubin
TP: Total protein
LYP: Percentage of lymphocytes
GLB: Globulin
WBC: White blood cells
AIB: Albumin
DBIL: Direct bilirubin
RDW: Red cell distribution width
HCT: Hematocrit
RBC: Red blood cell count
Cr: Creatinine
CK: Creatine kinase
ChE: Cholinesterase
ADA: Adenosine deaminase
Hb: Hemoglobin
IBIL: Indirect Bilirubin
MCHC: Mean corpuscular hemoglobin concentration
TBA: Total bile acid (TBA)
NONMEM: Nonlinear mixed-effects modeling (NONMEM)
AUC: Concentration–time curve
popPK: Population pharmacokinetic
EHRs: Electronic health records
HGB: Haemoglobin concentration
MCV: Mean cellular volume
RBC: Red blood cell count
