# Supplementary figures and images for: Predicting quetiapine dose in patients with depression using machine learning techniques based on real-world evidence
Source: Ann Gen Psychiatry. 2024 Jan 6;23:5. doi: 10.1186/s12991-023-00483-w (PMC10771703; doi:10.1186/s12991-023-00483-w)

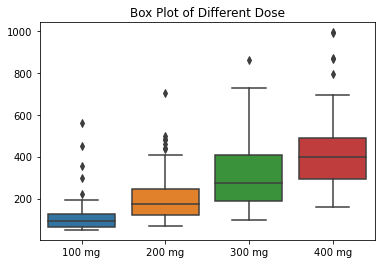

Supplement: Supplementary file 1 — Additional file 1. Figure S1. Boxplot of different doses. [file 12991_2023_483_MOESM1_ESM.png]
