# Supplementary material for: Predicting quetiapine dose in patients with depression using machine learning techniques based on real-world evidence
Source: Ann Gen Psychiatry. 2024 Jan 6;23:5. doi: 10.1186/s12991-023-00483-w (PMC10771703; doi:10.1186/s12991-023-00483-w)
Supplement: Supplementary file 2 — Additional file 2. Table S1. Parameters of nine models. [file 12991_2023_483_MOESM2_ESM.docx]

**Supplementary Table S1**

| Model | Parameters |
| --- | --- |
| XGBoost | param_grid = {  'learning_rate': [0.01, 0.05, 0.1, 0.15, 0.2],  'max_depth': [3, 4, 5, 6, 7],  'n_estimators': [100, 200, 300, 400, 500],  'subsample': [0.5, 0.7, 1.0],  'colsample_bytree': [0.5, 0.7, 1.0] ,  'reg_lambda': [1, 5, 10, 15] ,  } |
| LGBM | param_grid = {  'learning_rate': [0.01, 0.05, 0.1, 0.15, 0.2],  'max_depth': [3, 4, 5, 6, 7],  'iterations: [100, 200, 300, 400, 500],  'subsample': [0.5, 0.7, 1.0],  'colsample_bytree': [0.5, 0.7, 1.0] ,  'reg_lambda': [1, 5, 10, 15] ,  } |
| RF | param_grid = {  'n_estimators': [100, 200, 300, 400, 500],  'max_depth': [ 3, 5, 7, 8, 10],  'max_features': [ 0.25, 0.5, 0.75, 1],  'min_samples_split': [2, 5, 7,10]  } |
| GBDT | param_grid = {  'n_estimators': [100, 200, 300, 500],  'learning_rate': [0.01, 0.05, 0.1, 0.2],  'max_depth': [3, 5, 7, 10],  'min_samples_split': [2, 5, 10]  } |
| SVM | param_grid = {  'C': [0.1, 1, 10, 100, 1000],  'gamma': [1e-3, 0.01, 0.1, 1, 10]  } |
| LR | param_grid = {  'penalty': ['l1', 'l2'],  'C': [0.1, 1, 10],  'solver': ['linear_lstsq', 'lbfgs', 'cd'],  } |
| ANN | param_grid = {  'hidden_layer_sizes': [(50,50), (50,100), (100,50), (100,100), (100,200)],  'activation': ['relu', 'tanh'],  'solver': ['adam', 'sgd'],  'learning_rate': [0.01, 0.05, 0.1, 0.2],  'max_iter': [200, 500, 1000]  } |
| TabNet | param_grid = {‘max_epochs’:[50, 100, 200],  ‘patience’:[10, 50, 100,150],  ‘batch_size’=[64, 128, 256],  ‘virtual_batch_size’:[8, 32, 64]  } |
| DT | param_grid = {  'max_depth': [ 3, 5, 7, 10],  'min_samples_split': [2, 5, 10],  'min_samples_leaf': [1, 2, 3, 4]  } |
